# Aryl Hydrocarbon Receptor Role in Co-Ordinating SARS-CoV-2 Entry and Symptomatology: Linking Cytotoxicity Changes in COVID-19 and Cancers; Modulation by Racial Discrimination Stress

**DOI:** 10.3390/biology9090249

**Published:** 2020-08-27

**Authors:** George Anderson, Annalucia Carbone, Gianluigi Mazzoccoli

**Affiliations:** 1 CRC Scotland & London, Eccleston Square, London SW1V 1PB, UK; anderson.george@rocketmail.com; 2 Division of Internal Medicine and Chronobiology Laboratory, Department of Medical Sciences, Fondazione IRCCS “Casa Sollievo della Sofferenza”, San Giovanni Rotondo, 71013 Foggia, Italy; annalucia.carbone@gmail.com

**Keywords:** SARS-CoV-2, COVID-19, aryl hydrocarbon receptor, immune, cancer, melatonin, mitochondria, acetyl-CoA, treatment, racism

## Abstract

There is an under-recognized role of the aryl hydrocarbon receptor (AhR) in co-ordinating the entry and pathophysiology of the severe acute respiratory syndrome-coronavirus-2 (SARS-CoV-2) that underpins the COVID-19 pandemic. The rise in pro-inflammatory cytokines during the ‘cytokine storm’ induce indoleamine 2,3-dioxygenase (IDO), leading to an increase in kynurenine that activates the AhR, thereby heightening the initial pro-inflammatory cytokine phase and suppressing the endogenous anti-viral response. Such AhR-driven changes underpin the heightened severity and fatality associated with pre-existent high-risk medical conditions, such as type II diabetes, as well as to how racial discrimination stress contributes to the raised severity/fatality in people from the Black Asian and Minority Ethnic (BAME) communities. The AhR is pivotal in modulating mitochondrial metabolism and co-ordinating specialized, pro-resolving mediators (SPMs), the melatonergic pathways, acetyl-coenzyme A, and the cyclooxygenase (COX) 2-prostaglandin (PG) E2 pathway that underpin ‘exhaustion’ in the endogenous anti-viral cells, paralleling similar metabolic suppression in cytolytic immune cells that is evident across all cancers. The pro-inflammatory cytokine induced gut permeability/dysbiosis and suppression of pineal melatonin are aspects of the wider pathophysiological underpinnings regulated by the AhR. This has a number of prophylactic and treatment implications for SARS-CoV-2 infection and cancers and future research directions that better investigate the biological underpinnings of social processes and how these may drive health disparities.

## 1. Introduction

Although severe acute respiratory syndrome-coronavirus (SARS-CoV)-2 entry and symptomatology have been extensively investigated, significant prophylactic and treatment breakthroughs have yet to emerge. The angiotensin converting enzyme 2 receptor (ACE2r) seems crucial to SARS-CoV-2 viral entry, as was the case with the previous SARS-CoV-1 outbreak in 2002–2003, whilst a number of factors, including possibly nicotine [1], may act to modulate such entry. The ACE2r also underpins SARS-CoV-2 entry into other organs and tissues, contributing to the wider multisystemic pathophysiology driving both severity and fatality. The emerging extensive research on SARS-CoV-2 pathophysiology have emphasized the importance of the initial pro-inflammatory cytokine response, which positively correlates with subsequent symptom severity and fatality. Dysregulation of the immune responses underpin SARS-CoV-2 infection severity, including heightened neutrophil, macrophage and mast cell pro-inflammatory cytokine production, coupled to the suppression of the endogenous anti-viral response from CD8+ T cells, natural killer (NK) cells, and gammaDelta (γδ) T cells [2,3,4].

Although the importance of the initial rise in pro-inflammatory cytokines have been widely emphasized, there has been relatively little investigation of the common consequences arising, including (1) cytokine suppression of pineal gland melatonin and circadian dysregulation; (2) cytokine-induced gut permeability and dysbiosis with consequences for alterations in immune regulation and activation; and (3) cytokine induction of indoleamine 2,3-dioxygenase (IDO), thereby increasing kynurenine and kynurenine activation of the aryl hydrocarbon receptor (AhR) [5]. The current article reviews such processes and their implications for the understanding and treatment of SARS-CoV-2 pathophysiology. Notably, fatality seems primarily associated with advanced age and an array of pre-existing high-risk medical conditions, including obesity, type II diabetes, cardiovascular diseases (CVDs), autoimmune disorders, and cancers [6,7]. All of these pre-existing high-risk conditions have some association with suppressed pineal melatonin, increased gut dysbiosis/permeability and elevated kynurenine pathway activity, suggesting that pre-existing high-risk conditions may act to prime these downstream pro-inflammatory cytokine consequences, in turn indicating their relevance to raised levels of SARS-CoV-2 severity and fatality. 

**Figure 1:**
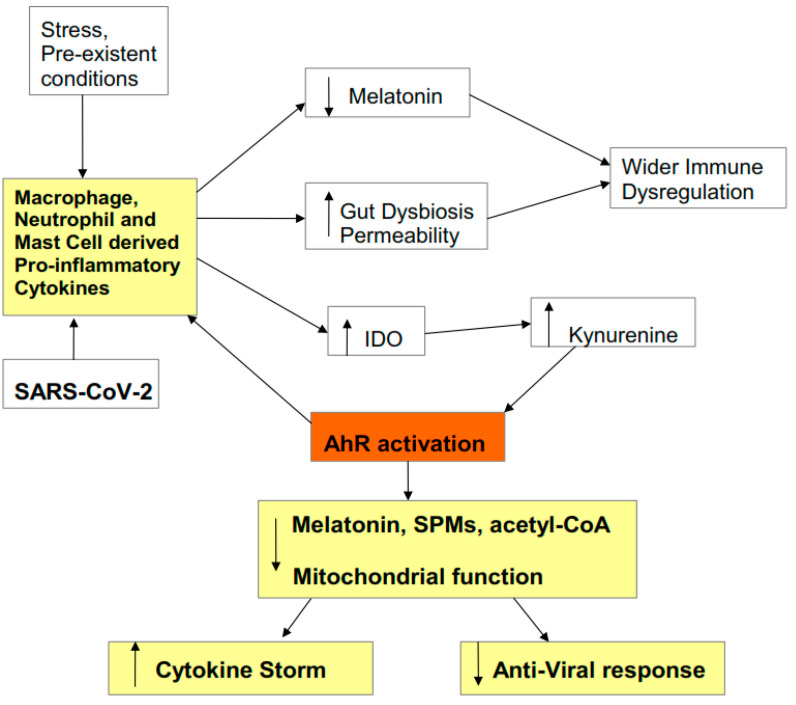
Pro-inflammatory cytokines decrease melatonin and increase gut permeability/dysbiosis, which contributes to immune dysregulation. Pro-inflammatory cytokines also increase indoleamine 2,3-dioxygenase (IDO), leading to kynurenine activation of the aryl hydrocarbon receptor (AhR). This potentiates the initial ‘cytokine storm’ and inhibits the endogenous anti-viral response, via the suppression of mitochondrial function, melatonin, acetyl-coenzyme A (CoA), and specialized pro-resolving mediators (SPMs). Stress, including racial discrimination stress, and pre-existent medical conditions prime and potentiate an elevated pro-inflammatory cytokine response, including via elevated kynurenine activation of the AhR.

As IDO utilizes tryptophan to produce kynurenine, there is a decrease in tryptophan availability for serotonin, N-acetylserotonin (NAS), and melatonin synthesis [5]. Consequently, an increase in kynurenine production and activation of the AhR is associated with a decrease in serotonin and melatonin. AhR activation, via cytochrome P450 (CYP) 1B1, may also lower melatonin availability, as will the AhR suppression of YWHAZ (14-3-3ζ/δ) [8], which is required to stabilize the first enzyme in the melatonergic pathway, aralkylamine N-acetyltransferase (AANAT). It is proposed that AhR activation and associated suppression of pineal and immune cell melatonin, coupled to the AhR’s suppression of acetyl-coenzyme A (CoA) and specialized pro-resolving mediators (SPMs), are crucial in driving the heightened cytokines underpinning the ‘cytokine storm’ as well as suppressing the endogenous anti-viral response. The AhR suppression of the anti-viral response parallels the immune suppression that is evident across most cancers. This provides a frame of reference that is supported by its capacity to incorporate wider bodies of data pertinent to SARS-CoV-2, including the association of Black Asian and Minority Ethnic (BAME) communities with heightened levels of severity and fatality [9]. The effects of a wide range of stressors have long been recognized to modulate virus susceptibility, severity, and recurrence [10,11], with both clinical and preclinical stressors shown to mediate many of their effects via kynurenine pathway upregulation. As such, the role of kynurenine activation of the AhR in driving SARS-CoV-2 pathophysiology also allows for the incorporation of discrimination stress-linked increases in severity and fatality in BAME communities. An overview of how pro-inflammatory cytokines modulate SARS-CoV-2 symptomatology is shown in Figure 1.

### The Aryl Hydrocarbon Receptor 

In humans, the aryl hydrocarbon receptor (AhR) is encoded by the AhR gene and was originally thought to function as a sensor of xenobiotic chemicals, being still widely referred to as the dioxin receptor. Its activation by the xenobiotic chemicals—aromatic (aryl) hydrocarbons—is how it derived its name. The AhR is long appreciated to mediate important effects via the upregulation of CYP1A, CYP1B1, and CYP1A2, as well as other metabolizing enzymes. Subsequent research showed the AhR to be activated by a wide array of endogenous, exogenous, and induced ligands, including FICZ, 2,3,7,8-tetrachlorodibenzo-p-dioxin (TCDD), and kynurenine. Accumulating evidence shows the AhR to have important roles in development, immunity, metabolism, and stem cell maintenance and a wide array of effects on diverse cellular processes and differentiation. 

The AhR is classified as a basic helix-loop-helix transcription factors and is usually present in an inactivated state in the cytosol, where it can be bound by a number of co-chaperones. Ligand binding leads to the dissociation of chaperones and the nuclear translocation of the AhR, where it dimerizes with the AhR nuclear translocator (ARNT), thereafter regulating a diverse array of genes, including those with a xenobiotic-responsive element (XRE). The AhR also induces its own repressor, the AhR repressor (AHRR), thereby inhibiting its transcriptional effects. 

The AhR is circadian regulated with distinct expression and effects over the circadian rhythm. AhR effects are further complicated by diverse effects in different cell types and following activation by different ligands. The AhR is widely expressed across organs and tissues, including in the lung.

## 2. SARS-CoV-2 Entry and Pathophysiology

The growing body of data on SARS-CoV-2 pathophysiology is now indicating important processes underpinning viral entry and symptomatology.

### 2.1. Entry 

**Figure 2:**
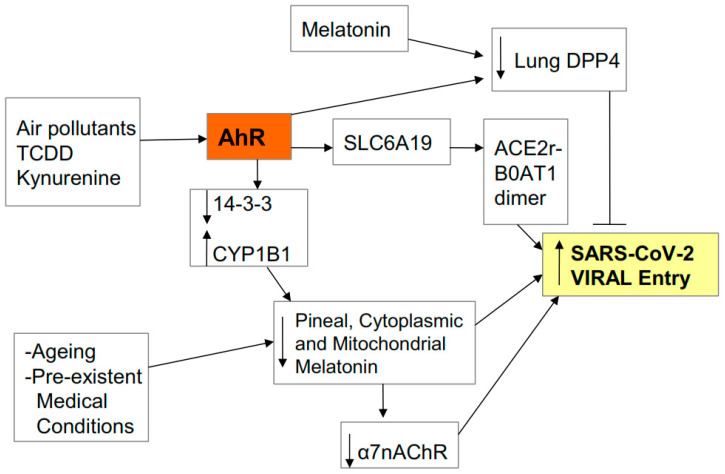
AhR activation by air pollutants, kynurenine or 2,3,7,8-tetrachlorodibenzo-p-dioxin (TCDD) regulates viral entry, including via SLC6A19 gene activation and B0AT1 induction. B0AT1 forms a dimer with the ACE2r which stabilizes the severe acute respiratory syndrome-coronavirus-2 (SARS-CoV-2) virus and greatly potentiates viral entry. Other dimers/partners of the ACE2r may form a similar function, including TMPRSS2 in the lung. The ACE2r and its partners require to be within lipid rafts, where the presence and/or activation of the α7nAChR in rafts inhibits viral entry. The putative beneficial effects of nicotine against viral entry seem mediated by an increase the levels and activation of the α7nAChR. Ageing, pre-existent medical conditions and AhR activation inhibit melatonin, thereby suppressing the melatonin induction of the α7nAChR. AhR activation, like melatonin, may suppress viral entry by decreasing viral entry via another receptor (lung DDP4).

The SARS-CoV-2 virus, like the 2002 SARS-CoV virus, gains entry via the ACE2r in pulmonary epithelial cells and in other tissues and organs. The ACE2r needs to be within lipid rafts and seems to need to form a dimer in order for SARS-CoV-2 to gain entry, including dimerization with the neutral amino acid transporter B0AT1 (encoded by SLC6A19) [12,13]. The ACE2-B0AT1 dimer may be especially important to non-lung viral entry, as B0AT1 does not seem to be constitutively evident in pulmonary epithelial cells, and where the host serine protease, TMPRSS2, may be a more important partner for the ACE2r [14]. As the pro-inflammatory cytokine-induction of IDO-kynurenine and consequent AhR activation induces B0AT1/SLC6A19 [15], this would suggest a role for AhR ligands in the regulation of SARS-CoV-2 viral entry, if not in the lung, then in the other organs/tissues in which B0AT1 is constitutively active. AhR ligands include air pollutants and the cigarette smoke constituent TCDD, as well as a wide array of endogenous and induced ligands, including kynurenine. The apparent protective effects of cigarette smoking in decreasing SARS-CoV-2 risk have been attributed to nicotine effects at the alpha 7 nicotinic acetylcholine receptor (α7nAChR), which is present in lipid rafts throughout the body, including in pulmonary epithelial cells, and affords protections against viral and bacterial lung infections [16]. However, the SARS-CoV-2 virus may also gain entry via the dipeptidyl peptidase-4 (DPP4) receptor, which is highly expressed in pulmonary epithelial cells and is inhibited by AhR activation, suggesting that TCDD may suppress viral entry via the lung, but enhance entry in other organs/tissues via B0AT1/SLC6A19 induction. It is also of note that melatonin can inhibit the DPP4 induction [16], suggesting that variations in melatonin production, invariably dramatically decreased in the very elderly, will also modulate viral entry via DPP4. (See Figure 2).

### 2.2. Pathophysiology

The primary pathophysiology of SARS-CoV-2 infection involves the dramatic upregulation of pro-inflammatory cytokines, induced by the activation of neutrophils, macrophages and mast cells, in what is often referred to as the ‘cytokine storm’. This includes increases in interleukin (IL)-1β, IL-6, C-reactive protein, and tumour necrosis factor (TNF) α [17]. This is evident in most respiratory infections and is usually followed within one week with a gradual increase in levels and activity of the endogenous anti-viral cells, viz CD8+ T cells, NK cells and γ δ-T cells. However, the activity of this anti-viral response is impaired in SARS-CoV-2 infection, with these suppressed cells showing evidence of ‘exhaustion’, which is classically associated with the immune-suppression evident in these cells within the tumour microenvironment. In the tumour microenvironment, CD8+ T cells, NK cells, and γδ-T cell ‘exhaustion’ arises from the release of kynurenine by cancer stem-like cells, which activates the AhR on these immune cells [18]. This would suggest that the suppressed/exhausted response of the endogenous anti-virals in SARS-CoV-2 infection may be mediated by increased AhR activation, paralleling their immune-suppression in the tumour microenvironment. 

### 2.3. Cytokine Storm Consequences

As noted, there are three common consequences of raised pro-inflammatory cytokines: viz pineal melatonin suppression, gut permeability/dysbiosis, and IDO/kynurenine/AhR activation (see Figure 1). These downstream cytokine consequences are important to SARS-CoV-2 pathophysiology. The loss of pineal melatonin dysregulates the circadian rhythm and suppresses melatonin’s anti-inflammatory and antioxidant effects. This is of some importance as melatonin acts to dampen the immune system at night, mediated by its optimization of oxidative phosphorylation (OXPHOS) [19,20]. Increased gut permeability leads to the release of lipopolysaccharide (LPS) in the circulation, which acts to dysregulate the immune response, as well as contributing to gut dysbiosis and therefore decreasing the epigenetic regulation of the immune system by the short-chain fatty acid butyrate, as evidenced across a host of diverse medical conditions [21]. The third ‘cytokine storm’ consequence is the induction of the IDO-kynurenine-AhR pathway, which, as noted, modulates both SARS-CoV-2 entry and pathophysiology. 

Data in SARS-CoV-2 patients show these three downstream consequences of the ‘cytokine storm’ to be evident, with patients showing increased gut permeability and gut dysbiosis [22], circadian dysregulation [23] and increased circulating kynurenine [24]. Given the impact of these three pro-inflammatory cytokine consequences on the immune response, an understanding of their role in SARS-CoV-2 entry and pathophysiology should allow for more targeted prophylactic and symptomatic treatment. 

### 2.4. AhR Regulation of Mitochondrial Metabolism

**Figure 3:**
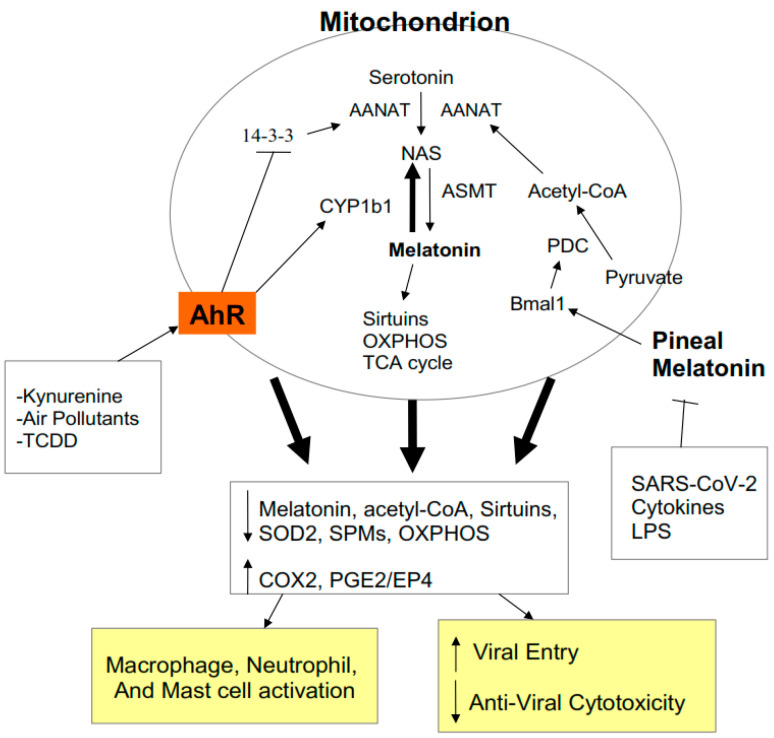
AhR activation contributes to alterations in mitochondrial metabolism as does suppressed pineal melatonin. By increasing CYP1B1 and suppressing 14-3-3, AhR activation lowers mitochondrial and cytoplasmic melatonin, thereby decreasing sirtuins and superoxide dismutase (SOD)2, contributing to suboptimal mitochondrial function. The decrease in circadian pineal melatonin in the aged, as well as from increased cytokines and gut permeability, lowers Bmal1 induction, which is crucial to upregulating pyruvate dehydrogenase complex (PDC) and its conversion of pyruvate to acetyl-CoA. Acetyl-CoA is a necessary co-substrate of AANAT and therefore for the initiation of the melatonergic pathway. The release of melatonin by cells of the ‘cytokine storm’ is necessary for their switch to a quiescent/phagocytic phenotype and this seem co-ordinated with SPMs induction. Acetyl-CoA is necessary for optimized oxidative phosphorylation (OXPHOS) and tricarboxylic acid (TCA) cycle ATP production. Acetyl-CoA also inhibits the COX2/PGE2/EP4 pathway that underpins exhaustion and is necessary for the increase in glycolysis that is required for anti-viral cells to become activated. The AhR and pineal melatonin act to regulate immune cell metabolism, activity and response to viral infection.

Alterations in metabolism are crucial to immune cell function. All immune cells have to upregulate glycolytic metabolism in order to become activated whilst also maintaining their levels of OXPHOS [5]. As such, factors acting to regulate OXPHOS and glycolytic metabolism can dramatically influence immune cell function. The AhR can regulate mitochondrial function by a number of mechanisms, including via the regulation of the melatonergic pathway. There is a growing appreciation of the role of the melatonergic pathway, including within mitochondria and the cytoplasm, in the regulation of mitochondrial function, which may be particularly important in immune cells [25]. The AhR can regulate the melatonergic pathway via a number of mechanisms, including 1) the induction of CYP1A1 and CYP1B1, with CYP1A1 increasing the metabolism of estrogen, whilst CYP1B1 leads to the ‘backward’ conversion of melatonin to its precursor, NAS, and thereby increasing the NAS/melatonin ratio [26]; and 2) AhR suppression of YWHAZ (14-3-3ζ/δ) [8], given that 14-3-3ζ/δ is necessary to stabilize the initial enzyme in the melatonergic pathway AANAT. As the autocrine effects of melatonin are necessary for immune cells to switch from an M1-like pro-inflammatory phenotype to an M2-like phenotype [27], AhR activation, via inhibition of the melatonergic pathway, would be expected to contribute to a prolongation of the ‘cytokine storm’. As immune cell activation requires the upregulation of glycolysis, the autocrine effects of melatonin act to inhibit glycolysis whilst enhancing OXPHOS, possibly in collaboration with specialized pro-resolving mediators (SPMs) [28]. Such alterations in macrophage, neutrophil and mast cell metabolism arising from AhR suppressed melatonin and SPMs will contribute to heightened immune-inflammatory activity by these cells during SARS-CoV-2 infection. (See Figure 3).

As noted, similar processes occur in the anti-viral cells, which contributes to the ‘exhaustion’ of these cells [18]. This seems to arise from the necessity of anti-viral cells to have optimized OXPHOS concurrent to the increased glycolysis that is required for their activation [29]. Consequently, the expected pattern during respiratory infection of increased macrophage/neutrophil/mast cell activation of the ‘cytokine storm’ gradually dampening as their activity contributes to an anti-viral response breaks down, resulting in the maintenance of the initial ‘cytokine storm’ and suppressed/exhausted anti-viral response, thereby leading to a pattern associated with SARS-CoV-2 severity and fatality [30]. AhR activation has a role in both aspects of the SARS-CoV-2 impacts on patterned immune responses.

The circadian rhythm driven by pineal melatonin release involves the ‘resetting’ of mitochondrial metabolism to OXPHOS in association with the upregulation of sirtuins and endogenous antioxidant enzymes, including superoxide dismutase (SOD)2 [31,32,33]. Pineal melatonin increases the circadian gene Bmal1, leading to pyruvate dehydrogenase kinase (PDK) inhibition, thereby disinhibiting the pyruvate dehydrogenase complex (PDC). PDC drives the conversion of pyruvate to acetyl-CoA, thereby increasing ATP production by the tricarboxylic acid (TCA) cycle and OXPHOS. Importantly, acetyl-CoA is also a necessary co-substrate for AANAT and the initiation of the melatonergic pathway. As such, night-time pineal melatonin acts to ‘reset’ mitochondrial metabolism, at least in part, via the upregulation of the TCA cycle, OXPHOS, and the mitochondrial melatonergic pathway, with AhR suppression of the melatonergic pathway attenuating these mitochondrial changes. It is via such changes in mitochondria and mitochondrial metabolism that the AhR acts to dysregulate patterned immune responses. (See Figure 3).

### 2.5. AhR and Pre-Existing High-Risk COVID-19 Medical Conditions

The role of the AhR in SARS-CoV-2 severity/fatality is supported by data regarding the pre-existing high-risk medical conditions for SARS-CoV-2 severity/fatality, including obesity, type II diabetes, CVDs, and old age. All these high-risk conditions are associated with elevated pro-inflammatory cytokines, gut permeability/dysbiosis, and kynurenine pathway activation [6], as well as decreased pineal melatonin production coupled to metabolic dysregulation [7]. The heightened kynurenine levels arising from such changes in high-risk conditions prime these conditions for SARS-CoV-2 severity via a pre-existent rise in AhR activation and the impact that this has on the immune response to SARS-CoV-2 infection. The heightened levels of stress and depression in these high-risk conditions, as well as stress/depression per se, will also increase kynurenine activation of the AhR. 

### 2.6. AhR and Stress 

The physiological consequences of stress include the activation of IDO and tryptophan 2,3-dioxygenase (TDO), contributing to an increase in kynurenine and kynurenine pathway products [34]. Psychosocial stress in children [35] and adults [36] increases immune-inflammatory responses and kynurenine, which contribute to the susceptibility to a number of medical conditions, including depression, neuropsychiatric disorders, and a number of cancers [37]. Classical conceptualizations of stress and depression have attributed such changes to the associated decrease in brain serotonin as well as to the neuroregulatory effects of kynurenine pathway products, such kynurenic acid and quinolinic acid [38]. However, there is a growing appreciation that even classical psychiatric disorders—such as depression [39,40], anxiety [41], and schizophrenia [42]—are powerfully determined by alterations in the kynurenine pathway and the AhR regulation of the immune system. The role of neuronal activity in mediating stress/depression has now shifted to viewing neuronal activity more as a form of ‘immune-to-immune’ communication [43]. Some of the effects of stress on the immune system are also mediated via an increase in gut permeability/dysbiosis [44], which, as noted below, also act to regulate the AhR. 

### 2.7. Stress and the Gut

**Figure 4:**
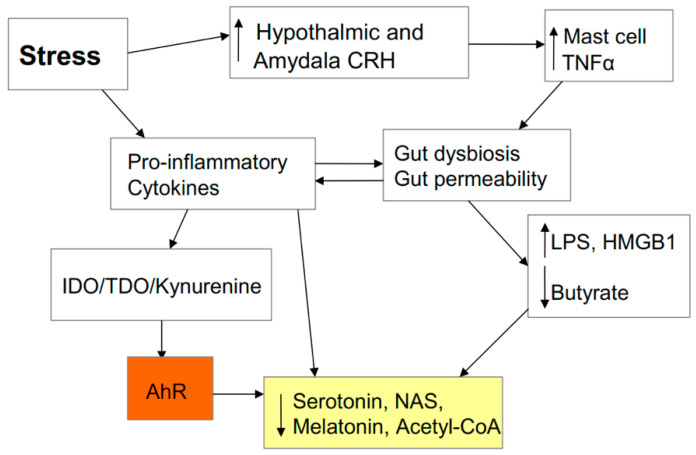
Stress increases hypothalamic and amygdala corticotropin-releasing hormone (CRH) release, thereby activating mucosal mast cells to release tumour necrosis factor (TNF) α, which increases gut permeability/dysbiosis. This leads to an increase in the toll-like receptor (TLR) 4 agonists lipopolysaccharide (LPS) and high-mobility group box (HMGB) 1, which decrease pineal melatonin production, thereby dysregulating the circadian regulation of optimized immune cell function. Decreased butyrate will also lower melatonin and acetyl-CoA production as well as contributing to suboptimal mitochondrial function. Stress, partly by increasing gut permeability/dysbiosis and pro-inflammatory cytokines, leads to an increase in the conversion of tryptophan to kynurenine, thereby lowering serotonin, N-acetylserotonin (NAS), and melatonin levels. Stress, in its many manifestations, is therefore intimately linked to pathways pertinent to the regulation of SARS-CoV-2 severity/fatality.

Preclinical and human data shows that psychological stress increases hypothalamic and amgydala corticotropin-releasing hormone (CRH) release [45]. Classically, CRH has been studied as a precursor for the hypothalamic-pituitary-adrenal (HPA) axis, leading to adrenal cortisol release. However, CRH also has independent effects, including activating mucosal mast cells to increase release of the pro-inflammatory cytokine, TNFα, which induces IDO and kynurenine and also acts on the gut epithelial cells to increase gut permeability. This is one aspect of stress that is likely to underpin the association of stress and stress-associated medical conditions with SARS-CoV-2, given the increased gut permeability/dysbiosis evident in SARS-CoV-2 patients [20]. Preclinical data support the role of kynurenine and immunity in the pathophysiology of stress/depression [46,47], including via alterations in the gut and in mitochondrial function [48], highlighting a link between kynurenine pathway induction and alterations in mitochondrial function. The effects of stress in the gut include a rise in circulating LPS and a decrease in butyrate as well as increased release of the alarmin, high-mobility group box (HMGB) 1 within exosomes. HMGB1 and LPS, activate toll-like receptor (TLR) 4, thereby contributing to an increase in pro-inflammatory cytokines [49,50]. Stress-induced alterations in the gut may therefore can be intimately linked to inflammatory cytokine production and the modulation of patterned immune responses, including via the AhR, as detailed below. As to whether racial discrimination stress contributes to the increased severity/fatality in BAME communities is discussed below. It is important to note that raised cytokines, by inducing IDO/TDO and kynurenine activation of the AhR, drive tryptophan down the kynurenine pathway and away from serotonin, NAS, and melatonin production. This has significant consequences for neuronal regulation and is important in how stress can drive depression, including as a consequence of gut permeability/dysbiosis. (See Figure 4).

## 3. AhR and Wider COVID-19 Pathophysiology

There is an exponential growth in data pertaining to SARS-CoV-2 pathophysiology. The above has highlighted under-explored consequences of the ‘cytokine storm’ and their priming by pre-existent conditions, upon which wider bodies of data may be integrated, including data on the role of coagulation and embolisms in SARS-CoV-2 fatalities.

### 3.1. AhR, Platelets, ROCK, and SARS-CoV-2 Severity/Fatality

Accumulating data indicate that ‘embolism emergence’ is a significant contributor to SARS-CoV-2 infection fatalities [51]. As AhR activation primes platelets for coagulation and aggregation, as well as increasing thrombin and fibrin (ogen) [52], clearly increased AhR activation will contribute to COVID-19 fatalities. As well as regulating the melatonergic pathway via CYP1B1 induction and 14-3-3ζ/δ suppression, the AhR also acts via the induction of sphingosine kinase (SphK)-induced sphingosine-1-phosphate (S1P) and S1P3 receptor (S1P3r) activation, thereby increasing the small GTPase, RhoA, and RhoA-associated kinase (ROCK) [53,54]. S1P modulates pulmonary epithelial cell infection responses [55], with RhoA/ROCK dysregulating the renin-angiotensin system and thereby driving the development of pulmonary embolisms [56]. Overall, AhR priming of platelets and RhoA-ROCK pathway induction in endothelial cells, thereby slackening tight junctions, increasing immune cell chemotaxis, and heightening inflammatory activity form the underpinnings to embolism formation [57]. 

**Figure 5:**
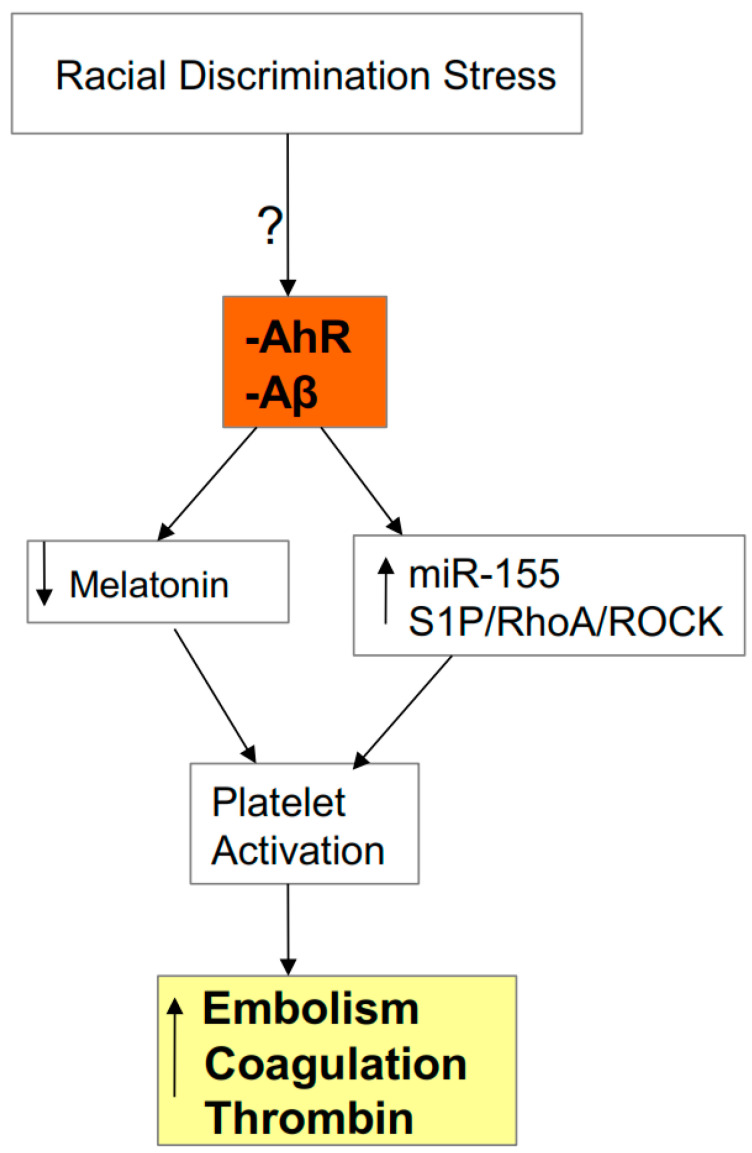
AhR activation and heightened Aβ levels in the elderly will contribute to platelet activation via decreased melatonin and upregulation of microRNA (miR)-155 and the S1P3r/RhoA/ROCK pathway. The resultant increase in coagulation, thrombin and new embolism formation contribute to SARS-CoV-2 severity/fatality. Racial discrimination stressors may contribute to such processes, given the heightened association of embolism-linked fatalities in African-American deaths during surgery for other types of medical conditions.

AhR ligands and antagonists will therefore have significant impacts on processes driving SARS-CoV-2 severity/fatality. Cytokine- and stress-induced kynurenine, as well as air pollutants and TCDD exposure, will contribute to embolism-associated fatality via the AhR activation and the induction of the S1P3 receptor/RhoA/ROCK pathway, including via AhR effects in platelets. The high levels of circulating amyloid-beta (Aβ) in elderly COVID-19 patients will also raise platelet RhoA levels and the prothrombin, pro-coagulation platelet phenotype [58], suggesting that the raised circulating Aβ levels in the elderly will contribute to SARS-CoV-2 severity and embolism-linked fatalities (see Figure 5). A number of common nutriceuticals, including green tea polyphenols, resveratrol, and vitamin B12, are AhR antagonists, suggesting that variations in their dietary intake may modulate SARS-CoV-2 severity (see Treatment section). 

### 3.2. AhR, Acetyl-CoA, COX2, and Specialized Pro-Resolving Mediators (SPMs)

The induction of SPMs is important to the resolution of immune inflammation. Interestingly, SPM regulation overlaps with the regulation of the melatonergic pathway, with both being upregulated by increased acetyl-CoA availability. SPM induction will also act to suppress the SphK1 and S1P/RhoA/ROCK pathway that underpin the emergence of SARS-CoV-2 embolisms [56]. Elevations in acetyl-CoA and Sphk1 allows acetyl-CoA to bind the sphingosine in SphK1 to form N-acetyl-sphingosine (N-ASph). N-ASph acetylates cyclooxygenase (COX) 2, thereby dampening COX2-induced inflammatory processes and upregulating SPMs [59]. In immune cells, N-ASph and acetylated-COX2 drive the shift from an M1-like pro-inflammatory phenotype to an M2-like phagocytic phenotype [59]. As acetyl-CoA is a necessary co-substrate for AANAT in the initiation of the melatonergic pathway, this indicates that the raised acetyl-CoA levels underpinning N-ASph production can be co-ordinated with AANAT stabilization and melatonergic pathway activation. As such, SPM induction may be intimately linked to cytoplasmic and mitochondrial melatonin production via increased acetyl-CoA and therefore with TCA cycle ATP, OXPHOS, and mitochondrial metabolism. Factors acting to dysregulate mitochondrial metabolism, and therefore acetyl-CoA levels, will therefore impact on the co-ordinated induction of the melatonergic pathway, N-ASph, and SPMs and therefore COX2 and immune phenotype regulation. 

This may be exemplified in macrophages where autocrine melatonin switches M-like pro-inflammatory macrophages to an M2-like phagocytic phenotype [25]. As to how SPMs and autocrine melatonin interact to downregulate activation associated glycolysis, whilst upregulating OXPHOS will be important to determine. It would seem likely that the acetyl-CoA stabilization of mitochondria AANAT is intimately linked to the regulation of mitochondrial sirtuins and SOD2, as well as the mammalina target of rapamycin (mTOR) pathway, which is important to glycolysis regulation [17,30]. However, the temporal and spatial interactions of melatonin with SPMs require clarification, including as to how these interactions are modulated during the SARS-CoV-2 ‘cytokine storm’. This will be important to determine, given that the COX2-PGE2 pathway, regulated by melatonin, N-ASph and SPMs, is an important driver of macrophage and neutrophil activation and migration, in association with cytoskeletal regulation driven by RhoA/ROCK and the RhoA/ROCK induction of COX2 [60]. As raised glucose levels, often evident in obesity/type II diabetes, activate macrophages via the RhoA/ROCK pathway [61], macrophages may be primed for heightened responsivity in these SARS-CoV-2 high-risk conditions. There is currently no direct data looking at the role of the AhR in the regulation of N-ASph, or indeed of the SPMs. Clearly, the AhR suppression of melatonin would dramatically alter such proposed acetyl-CoA driven interactions of SPMs and melatonin in the regulation of these key cells that drive the ‘cytokine storm’. 

**Figure 6:**
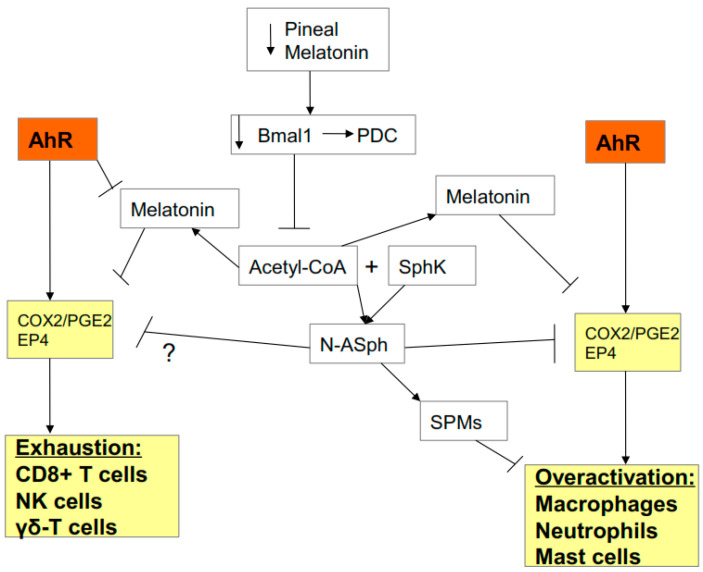
AhR-induced cyclooxygenase (COX) 2/PGE2/EP4 has differential effects in anti-viral cells vs. cells of the ‘cytokine storm’. Levels of acetyl-CoA may be significant determinants of AhR effects via the COX2/PGE2/EP4 pathway via acetyl-CoA using sphingosine from sphingosine kinase (SphK) to produce N-acetyl-sphingosine (N-ASph), which, like aspirin, acetylates and inhibits COX2. Acetyl-CoA, as a co-substrate for AANAT, also increases melatonin, which also inhibits this AhR-driven pathway. As such, acetyl-CoA may act to co-ordinate N-ASph and melatonin production and effects, with their differential consequences in NK cells and CD8+ t cells vs. macrophages, neutrophils, and mast cells. This would suggest that variations in acetyl-CoA in these cells will determine their differential activation in SARS-CoV-2 infection, as well as in cancers. The suppression of melatonin by the AhR in all of these cell types will contribute to their dysregulation in SARS-CoV-2 infection, whilst the suppression of pineal melatonin, via a decrease in Bmal1-induced PDC and therefore acetyl-CoA production, will be important in driving the dysregulated immune response in the aged population as well as in those with high-risk, pre-existent medical conditions. N-ASph can also increase SPMs in macrophages, which, along with autocrine melatonin, switches activated macrophages to a more quiescent phagocytic phenotype.

It should be noted that SPMs suppress the AhR and AhR-induced genes, including COX2 [62]. Many AhR effects are mediated via COX2 induction [60], including in air-pollutant-activated macrophages [63] and in dendritic cells where AhR activation-induced COX2 is crucial to the induction of the high-inflammation and autoimmune-associated Th17 cells [64]. COX2 is also a significant regulator of NK cell function, both directly and via COX2 effects in dendritic cells [65]. As such, acetyl-CoA levels, via N-ASph acetylation of COX2, can have dramatic effects on pro- vs. anti-inflammatory activity in immune cells, with concurrent impacts on levels of melatonin production, release, and autocrine effects that modulate the immune cell phenotype, with acetyl-CoA also driving paths that optimize mitochondrial metabolism and suppress AhR activity and effects. Previous data indicating both pro- and anti-thrombotic effects of COX2 [66] may also be attributed to its distinct effects that are dependent upon its state of acetylation, and therefore on the levels of acetyl-CoA and N-ASph. (See Figure 6).

Kynurenine activation of the AhR induces COX2-PGE2 and EP4 receptor activation in NK cells, leading to the suppressed/‘exhausted’ NK phenotype that is typical in the tumour microenvironment [67], and which is paralleled in severe SARS-CoV-2 infection. The COX2-PGE2 pathway inhibits NK cells migration, cytotoxic effects and IFNγ secretion [68], with similar effects in CD8+ T cells [69] and γδ T cells [70]. As to whether acetyl-CoA in NK cells or CD8+ T cells modulates N-ASph production to increase acetylated-COX2, thereby decreasing PGE2 and EP4 receptor activation will be important to determine. As acetyl-CoA is linked to mitochondrial metabolism and the initiation of the melatonergic pathway, alterations in melatonin, TCA cycle, and OXPHOS will be intimately associated with N-ASph and acetylated-COX2 in the regulation of NK cells and CD8+ T cell and γδ T cell anti-viral and anti-cancer responses. Although the activation and cytotoxicity of these cells requires the upregulation of glycolysis, the maintenance of OXPHOS is crucial to the prevention of a suppressed/’exhausted’ NK cell phenotype. As PGE2 production by nearby cells, including macrophages [71], may allow neighbouring cells to suppress NK cells and CD8+ T cells, it is likely that inhibition of COX2-PGE2 more widely may be of use. This has been the rationale for the extensive use of COX2 inhibitors in cancer treatments. 

The roles of different SPMs over the course of NK cell and CD8+ T cell activation/deactivation have still to be clarified. SPMs have significant immune-regulatory functions relevant to COVID-19 and cancer pathophysiologies. The RvE1 receptor, CMKLR1, is expressed on NK cells, where its activation leads to NK cell attraction and activation that resolves lung inflammation [72]. However, by acting to dampen macrophage/neutrophil/mast cell activation, RvD1 and RvE1 also decrease the chemoattraction and activation of NK cells and CD8+ T cells, under some experimental conditions [73]. The temporal regulation of SPMs across different immune cells over the course of SARS-CoV-2 infection will be important to determine. SPM regulation may also be important in how pre-existent high-risk conditions for SARS-CoV-2 fatality mediate their susceptibility. Weight loss in obese patients increases neutrophil RvE1 release two-fold [74]. This seems of importance as a significantly increased RvE1 dose is required to dampen inflammation in the neutrophils of type II diabetic patients [75]. Such data indicate a role for pre-existent high-risk medical conditions–induced variations in SPMs in the suboptimal initial ‘cytokine storm’ and later anti-viral response evident in these patients. Pre-existent, cytokine-, or stress-induced increase in gut dysbiosis/permeability may be relevant to this, given that gut permeability-induced HMGB1 suppresses the RvD1 resolution of activated neutrophils [76]. Chronic heart failure patients show a decrease response to RvD1 and RvD2 in activated CD8+ T cells, mediated by a decrease in the GPR32 receptor [77]. Such data highlight how ongoing medical conditions modulate SPM levels and the SPM regulation of the immune response, with consequences that partly arise from the metabolic dysregulation in immune cells. 

Dendritic cells are important regulators of patterned immune responses, and data in these cells also highlight the importance of mitochondrial metabolism in determining cellular function. Acetyl-CoA carboxylase (ACC) leads to the irreversible carboxylation of acetyl-CoA, with AhR activation increasing ACC via Synphilin-1 degradation [78]. The suppression of sirtuin-1 in dendritic cells also increases ACC levels and decreases acetyl-CoA, with consequent alterations in mitochondrial metabolism that lead to dendritic cells inducing deficient anti-viral responses [79]. ACC blockade in this study led to dendritic cell metabolic reprogramming that ameliorated mitochondrial dysfunction and restored a more appropriate anti-viral response [79]. Such data highlights the important role that acetyl-CoA and its regulation by ACC have in determining and fine-tuning mitochondrial function [80], and the impact that alterations in mitochondrial function have on the immune regulatory responses of dendritic cells. This is also relevant to ageing, as ACC inhibition prevents accelerated ageing in a preclinical model [81], suggesting that the association of ageing with both cancer and SARS-CoV-2 infection fatality may be linked to suppressed acetyl-CoA levels in dendritic cells and the consequences that this has for patterned immune responses, including within cells determining anti-viral and anti-cancer responses. It should be noted that increased ACC will also suppress the melatonergic pathway, which will also have consequences for the regulation of immune cells, SPMs and sirtuins. 

Overall, alterations in the regulation of acetyl-CoA will have impacts on AhR effects, acetylation levels of COX2, SPM levels, and melatonergic pathway activity, with consequences that will determine mitochondrial function in immune cells and associated variations in patterned immunity, thereby modulating inflammation as well as anti-viral and anti-cancer responses. 

### 3.3. AhR, COX2, SPMs, Acetyl-CoA, and miR-155

Although the ‘cytokine storm’ increases LPS and pro-inflammatory cytokines that will raise miR-155 levels [82], there is no data pertaining to miR-155 following SARS-CoV-2 infection. miR-155 has a number of effects important to SARS-CoV-2 pathophysiology and anti-viral/cancer responses, including Bmal1 suppression [83], and therefore suppression of the Bmal1/PDC/OXPHOS/TCA cycle linked to increased acetyl-CoA production. Elevations in miR-155 are evident in COVID-19 high-risk, pre-existent conditions, including obesity [84], type II diabetes [85], hypertension and CVDs [86], and most human cancers [87]. Raised miR-155 levels promotes inflammatory cytokine production in macrophages and microglia, in association with a decrease in sirtuin-1 [88], linking increased miR-155 to a heightened and prolonged ‘cytokine storm’, including from a primed elevation of miR-155 in high-risk conditions. Preclinical data show the raised miR-155 levels in immune cells to accelerate ageing and decrease longevity via an increase in aerobic glycolysis and associated induction of pro-inflammatory processes [89]. The miR-155 inhibition of sirtuin-1 may be important to ageing via ACC upregulation and acetyl-CoA suppression [79], suggesting that miR-155 will associate with lower N-ASph, melatonin and acetylated-COX2. Melatonin increases sirtuins and decreases miR-155 levels [90,91], highlighting the importance of suppressed melatonin, including pineal and immune-derived melatonin. 

miR-155 has been proposed as ‘master regulator’ of dendritic cell function and of the role of dendritic cells in modulating patterned immune responses. miR-155 over-expression enhances the the initial cytotoxicity of CD8+ cells [92] and NK cells [93]. Such data indicate the importance of miR-155 in the raising the glycolytic metabolism crucial to immune cell activation. However, as the maintenance of OXPHOS is necessary for prolonged immune activation in these cells, the suppression of OXPHOS mediated by miR-155 upregulation of COX2-PGE2 and EP4 receptor activation, coupled to its suppression of Bmal1-linked OXPHOS will contribute to ‘exhaustion’. Data in CD8+ T cells support this, with the long-term persistence of exhausted CD8+ T cells being maintained by raised miR-155 levels during chronic infection [94]. It should be noted that miR-155 has distinct effects in different cells types, as indicated by its differential effects on an array of standardized mRNAs in B-cells, T cells, dendritic cells, and macrophages [95]. The evolved interactions of melatonin with miR-155 in different cell types may suggest that melatonin is a more viable treatment regulator of miR-155 than miR-155 targeted pharmaceuticals. However, AhR activation, by suppressing acetyl-CoA and melatonin whilst increasing COX2/PGE2 and EP4 receptor activation, will act to suppress any miR-155 associated initial activation of anti-viral cells, whilst potentially augmenting miR-155 induced activation of macrophages, neutrophils and mast cells in the ‘cytokine storm’. The interactions of miR-155 and other miRNAs with variations in mitochondrial metabolism, acetyl-CoA levels, and melatonergic pathway activity will be important to determine in individual immune cell types and in the interactions of different types of immune cells.

To date, there is no data on the interactions of inflammation resolving SPMs and inflammation-inducing miR-155. However, indirect data indicates that this interaction may be of some importance. miR-155 binds COX2 and induces COX2 reporter activity, whilst maintaining COX2 mRNA stability [96]. The high similarity of COX2 and miR-155 effects in cancers [97], would indicate that the miR-155 upregulation of COX2 is an important aspect of miR-155 driven changes. Consequently, N-ASph acetylation of COX2 will modulate/inhibit the diverse effects of miR-155, in conjunction with increasing SPM production. Clearly, N-ASph and SPM effects on miR-155/COX2 in CD8+ T cells, NK cells, and γδ-T cells will be important to determine, as will their suppression of platelet activation [98], where different SPMs can have differential effects [99]. Pro-resolving ALX/FPR2 receptors, activated by Lipoxin-A4, are present on NK cells where their activation regulates NK activity [100]. Overall, AhR effects will be significantly modulated by the interactions of acetyl-CoA levels, N-ASph, melatonin, miR-155, and COX2-PGE2 with consequences for SPM induction and anti-viral/cancer cell OXPHOS, glycolysis, and associated cytotoxicity. 

### 3.4. Gut Dysbiosis: Interactions with Acetyl-CoA, COX2, SPMs, and AhR

As sodium butyrate epigenetically suppresses COX2-PGE2 via its inhibition of HDAC5/6 [101], this would suggest a role for gut dysbiosis in the regulation of miR-155 and AhR modulation of the immune response to the SARS-CoV-2 virus. Butyrate also regulates the mRNA binding protein, HuR, to decrease COX2 mRNA and protein expression [102]. Such data indicates a role for variations in the gut microbiome in the modulation of COX2-PGE2 effects on the immune response, including directly and via other cells, on CD8+ T cells, NK cells and γδ T cells. Butyrate can also be converted to acetyl-CoA (via acyl-CoA synthetase), indicating that gut-microbiome-derived butyrate may modulate N-ASph levels, acetylated-COX2, SPMs, and the melatonergic pathway via its conversion to acetyl-CoA [103]. Such data would indicate a gut dysbiosis relevant modulation of these crucial cells in cancers and SARS-CoV-2 infection. Butyrate also inhibits cells involved in the ‘cytokine storm’, including macrophages and neutrophils [104], with its inhibition of COX2-driven attachment of monocytes to endothelial cells [105], indicating an impact of butyrate on embolism formation and atherosclerosis [106]. In the lung, butyrate suppresses HMGB1 induction to infection, suggesting that it will act to prevent the HMGB1 inhibition of RvD1 and the RvD1 resolution of activated neutrophils [76]. It has been recently proposed that the effects of gut dysbiosis are crucially mediated by its regulation of systemic mitochondrial function, especially in immune cells [44]. The data above would suggest that this is partly driven by butyrate’s regulation of acetyl-CoA, and the consequences that this has for the AhR, COX2, SPMs, and melatonergic pathways. 

## 4. Integrating Racial Discrimination into SARS-CoV-2 Pathophysiology

The increased risk of SARS-CoV-2 infection severity/fatality in people from BAME communities has been attributed to a number of factors, including decreased vitamin D, and therefore suboptimal immune responses; racism-linked over-crowding, thereby increasing virus exposure and viral load; occupational bias toward jobs involving interpersonal, service interactions, such as bus drivers and care workers; and domestic dwellings in areas of high air pollutants, which may prime for lung infection. All of these factors are likely of some relevance and will contribute to the effects of how racial discrimination stress will modulate SARS-CoV-2 severity and fatality, at least in part via an increased risk of the pre-existing, high-risk medical conditions such as obesity, type II diabetes, CVDs, and hypertension. However, the increased risk of the BAME communities to severe and fatal SARS-CoV-2 infection is still evident when these factors have been taken into account [7], suggesting perhaps more direct effects of racial discrimination stress on biological processes underpinning SARS-CoV-2 pathophysiology. 

A plethora of data shows relatively increased levels of pro-inflammatory cytokines in African Americans, including in studies focussing on men, women, youths, the elderly, and in breast exudates as well as after the Trier Social Stress Test [107]. Where investigated, these studies show positive correlations of cytokines with reported racial discrimination experiences [107,108]. Such raised immune-inflammatory data are often utilized to explain the health disparities that exist between African Americans and European Americans and may be coupled directly to an accelerated ageing, as indicated by decreased telomere length in immune cells [109] or indirectly via the consequences of increased levels of obesity, type II diabetes, hypertension, CVDs, and cancers in African Americans. This work has typified an immune response where a heightened inflammatory activation, but suppressed anti-viral response, is evident under conditions of social disadvantage [110]. As noted above, accelerated ageing may be driven by increased ACC [81], thereby suppressing acetyl-CoA availability for N-ASph, SPMs, and melatonin production. This may be associated with an increase in miR-155, which is evident in stress/depression [111] and contributes to driving alterations in levels and patterning of immune activity that parallels those in ageing. 

Such data also parallels similar data regarding socio-economic status and is usually described as a stress-associated increase in ‘weathering’. However, as with COVID-19, there has been little investigation of the direct downstream consequences of racial discrimination stress–induced cytokines and any impact this may have on suppressed pineal melatonin, gut permeability/dysbiosis, and kynurenine pathway activation. Pineal melatonin urine metabolites are decreased in African Americans in correlation with raised night-time blood pressure [112], whilst an increase in LPS is evident in African Americans vs. European Americans in some studies, with LPS further increasing TNFα in obese African-American women [113]. There is no meaningful data on variations in the kynurenine pathway across ethnic background and none of these three downstream consequences of raised pro-inflammatory cytokines have been investigated as to their relationship with racial discrimination stress. 

**Figure 7:**
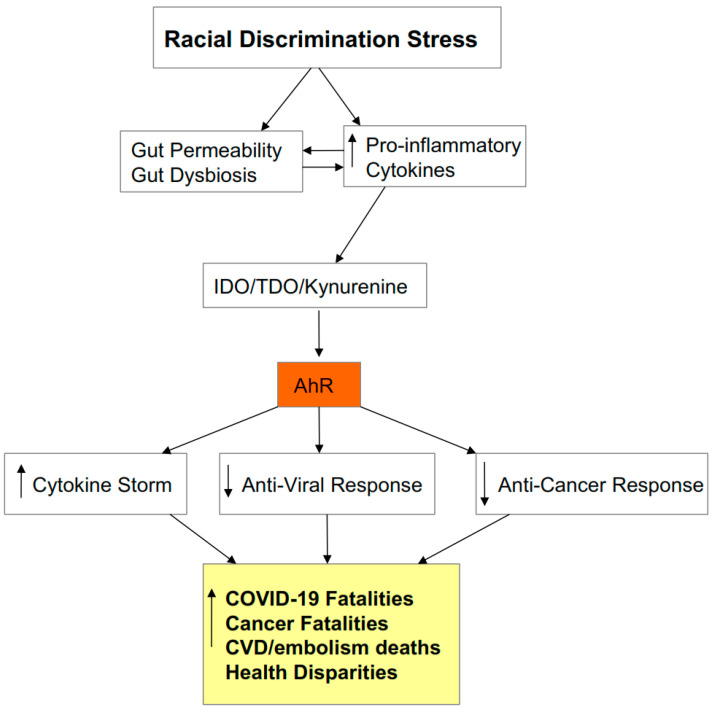
Racial discrimination is associated with increased basal and induced pro-inflammatory cytokines, driving IDO induction of kynurenine, and thereby leading to AhR activation that acts to increase macrophage and neutrophil inflammatory responses, whilst decreasing anti-viral and anti-cancer cell responses. The increased risk of CVD and embolism induced death in African Americans will be contributed to by AhR priming of immune cells and platelet responses. Racial discrimination increases the risk of deaths across many medical conditions and drives the health disparities that are most documented in the US, in ways that involve such processes. Racial discrimination acts via these processes to increase an array of other medical conditions, including type II diabetes that contribute to suboptimal immune responses to viruses as well as increasing cancer susceptibility. The racial discrimination links to gut permeability/dysbiosis require further investigation but are highly likely to contribute to immune dysregulation, given the increase in pro-inflammatory cytokines arising from racial discrimination stress.

Given the SARS-CoV-2-driven pathophysiology outlined above, the implications of this data for the raised severity/fatality levels in the BAME community are clear. Although increased exposure to air pollutants associates with the increased prevalence and severity of SARS-CoV-2 infection in African Americans [114], it would seem highly likely that racial discrimination experiences, like other high-risk conditions, will prime the immune response for more detrimental consequences to viral infection, whilst also suggesting that this will impact on cancer severity and poorer cancer outcomes in African Americans. Paralleling the effects of air pollution, the effects of racial discrimination can be seen to be acting via cytokine-induced IDO and kynurenine production, leading to AhR activation. As to whether the putative effects of discrimination stress act via AhR-RhoA/ROCK-driven coagulation and embolism formation in driving the increased fatality in BAME communities will be important to determine. Broader bodies of data show African Americans have an increased risk of pulmonary and venous embolisms after a variety of different operations for non-related conditions [115,116,117], possibly indicative a racial discrimination stress–potentiated risk of thrombo-embolisms and suggesting effects that are relevant not only to COVID-19 fatalities. (See Figure 7).

As AhR activation suppresses the anti-cancer effects of CD8+ T cells, NK cells, and γδ-T cells [16], it is notable that African Americans have an elevated risk of an array of different cancers as well as poorer cancer outcomes [118,119]. Whether discrimination-stress-induced kynurenine and increased air pollutant exposure contribute to such health disparity statistics via AhR activation requires investigation. Single nucleotide polymorphism (SNP) in ARNT, the nuclear translocator partner of the activated AhR, increases the risk of an array of immune-mediated diseases [120], highlighting the importance of the AhR-ARNT dimer-induced transcriptions in the modulation of immune-associated pathology. Numerous studies have highlighted variations in a number of SNPs in different genes across ethnicity to explain such differences. However, it may be of note that a comparison of influenza infection in children under five years of age, showed no differences in levels of illness severity between African Americans and European American children [121], at an age before racial discrimination stress is understood, but where SNP influences would still be expected to occur. 

## 5. Treatment Implications

A number of novel treatment implications emerge based on SARS-CoV-2 pathophysiology and an appreciation of the important role of the AhR in co-ordinating changes in patterned immune responses. The roles of nutriceuticals may be important, given their more realistic application as a prophylactic and in the modulation of racial discrimination stress and its impact on risk and pathophysiology of SARS-CoV-2 infection and an array of cancers.

### 5.1. AhR Antagonists

A plethora of nutriceuticals and pharmaceuticals have been shown modulate the IDO-kynurenine-AhR, including resveratrol, curcumin, and rosiglitazone [122]. These authors also highlight the potential direct effects of SARS-CoV-2 on AhR activity, which will be important to determine. However, the wider effects of vitamin B12/folic acid and especially green-tea-derived polyphenols in AhR regulation that are relevant to the ‘cytokine storm’ and the regulation of endogenous anti-viral responses are worth highlighting.

#### 5.1.1. Vitamin B12 and Folic Acid

Vitamin B12 and folic acid directly bind to the AhR, thereby suppressing its activation by endogenous and exogenous ligands [123]. Vitamin B12 is also a significant inhibitor of the SARS-CoV-2 protease, Mpro [124], suggesting that it will have impacts on both SARS-CoV-2 virus entry and symptoms. Optimizing vitamin B12 increases NK cell cytotoxicity [125]. 

Preclinical data shows the AhR to be a major mediator of obesity and liver steatosis, which can be reversed by AhR antagonism [126], implicating heightened AhR activity in the association of obesity and type II diabetes in COVID-19 and cancer risk and fatality. However, of the nutriceuticals, AhR antagonism by green tea has been most extensively investigated. 

#### 5.1.2. Green Tea Polyphenols

Green tea has a number of polyphenols, including epigallocatechin gallate (EGCG), which inhibits the AhR [127], suggesting the utility of EGCG in the inhibition of the AhR modulation of SARS-CoV-2 entry and pathophysiology and in the prevention of the immune suppression evident in most cancers. Notably, three of the polyphenols in green tea—EGCG, epicatechingallate, and gallocatechin-3-gallate—bind and inhibit the main SARS-CoV-2 protease, Mpro [128], indicating wider impacts of green tea polyphenols on virus entry. Bioactive molecules from teas can show greater binding to Mpro than some of the widely used, repurposed drugs, including Lopinavir [129]. Notably, EGCG has been found to exert anti-viral effects against a range of different virus types [130]. 

As also occurs in cancers, SARS-CoV-2 increases myeloid-derived suppressor cells (MDSCs), which correlates with a decrease in perforin-expressing NK cells and increased infection severity [131]. MDSCs may have a role in reducing inflammation, although coupled to the suppression of endogenous anti-viral cells [132]. Although not investigated in SARS-CoV-2 infection, EGCG decreases MDSC in cancers, thereby attenuating MDSC-driven immune suppression and increasing CD8+ T cells levels [133]. As EGCG inhibits the AhR, it would be expected to increase CD8+ T cells and NK cells cytotoxicity. This is further supported by EGCG’s enhancement of CD8+ T cell anti-cancer immunity [134]. EGCG may therefore optimize endogenous anti-viral responses by a number of means. 

As well as by inhibiting the AhR, the green tea polyphenols’ inhibition of monoamine oxidase [135], will lead to an increase in serotonin availability for the melatonergic pathway. EGCG also increases 14-3-3 [136] and therefore the stabilization of AANAT, which is important in the initiation of the melatonergic pathway, whilst its inhibition of glycolytic metabolism and induction of OXPHOS indicates a significant role in mitochondrial metabolism [137,138]. EGCG consequently shifts macrophages to an M2-like phenotype and decreases neutrophil levels and activation in lung sepsis [139], suggesting that it may dampen the initial ‘cytokine storm’ in COVID-19 and the longer-term lung injury arising from excessive neutrophil and macrophage activation [140]. Some of the effects of green tea are mediated via tannic acid, which inhibits pyruvate kinase isoenzyme M2 (PKM2), which is essential in aerobic glycolysis [141]. NK-cell-mediated cancer apoptosis by HMGB1 is via the inhibition of PKM2 [142], with PKM2 crucial to the expression of the immune checkpoint factor PD-L1 in CD8+ T cells and tumours [143], highlighting the importance of alterations in metabolism in regulating plasma membrane receptors and their ligands. PD-1 inhibitors are widely used in cancer treatments and have been proposed as a COVID-19 treatment [144]. 

It should also be noted that any hepatotoxicity associated with green tea intake is prevented by melatonin [145], suggesting utility of combining melatonin and EGCG in SARS-CoV-2 treatment. Melatonin also has proposed benefits in SARS-CoV-2 infection regulation [146].

### 5.2. Melatonin

Data on over 11,000 people tested for the presence of the SARS-CoV-2 virus indicate that the taking of melatonin decreases the risk of infection [147], possibly suggesting a role for variations in melatonin in the regulation of viral entry. This supported by recent data in a sample of 18,000 adults, showing melatonin to decrease the risk of SARS-CoV-2 infection by 64% [148]. Given the role of pineal and immune-cell-released melatonin in the modulation of immune cells, coupled to melatonin’s safety and anti-inflammatory and antioxidant properties, it is clear that melatonin would have prophylactic and treatment utility in the management of SARS-CoV-2 infection, including via its induction of the alpha 7 nicotinic receptor (α7nAChR) [5], as well as its dampening of macrophage activity via the activation of the nuclear retinoid-related orphan receptor α (RORα) [149]. Melatonin also protects endothelial cells directly, including from glucose dysregulation [150], and dampens platelet activation, suggesting protection against coagulation and embolism formation that contribute to many COVID-19 fatalities. 

### 5.3. ROCK Inhibitors

As many of the pathophysiological consequences of AhR activation are mediated via the activation of the S1P3r/RhoA/ROCK pathway that drive platelet activation and endothelial-driven changes leading to the emergence of embolisms, ROCK pathway inhibition is another treatment option. The only pharmaceutical ROCK inhibitor, Y27632, is approved in few countries, where it is exclusively applied locally to the eye and has a level of toxicity that would make it impractical in current COVID-19 management [151,152]. However, a number of substances may have secondary effects that suppress the S1P3r/RhoA/ROCK pathway, including nutriceuticals such as curcumin [153], suggesting their possible utility. 

### 5.4. IDO Inhibitors

IDO inhibition is an upstream target of AhR activation by kynurenine. IDO inhibitors have been extensively developed for cancer treatment. However, disappointing phase I/II trials have led to their utilization only as adjunctives to other cancer treatments, whilst its effects on the influenza virus do not indicate anti-viral utility [154]. 

### 5.5. Nimesulide

The B0AT1 inhibitor Nimesulide has been proposed as a treatment to decrease the entry of SARS-CoV-2 via the ACE2-B0AT1 dimer [10]. This will be important to investigate and may be relevant to SARS-CoV-2 entry in many organs following AhR-induced SLC6A19 [13].

### 5.6. Prophylaxis

On the basis of the above the following prophylactic treatment could be recommended:

Melatonin, 2–10 mg once nightly; green tea/EGCG at least twice daily as a drink or supplement; and nicotine patch/gum to increase nicotine activation of the α7nAChR. Optimization of vitamins, especially vitamin B12. As the inhibition of adenosine is an important treatment target to increase NK cell and CD8+ T cell cytotoxicity in cancers, caffeinated green tea may be more useful, as indicated by data in COVID-19 CD8+ T cells [155]

### 5.7. Treatment

Melatonin 5–8 mg/kg i.v. until ‘cytokine storm’ is dampened. An ongoing Phase II clinical trial should better clarify high dose melatonin utility [156]. Concurrent use of EGCG, which may be increased to optimize anti-viral responses via AhR inhibition. Nicotine patch may provide additional protection via α7nAChR activation, which dampens macrophage and neutrophil activation. High vitamin B12 dose to add to AhR inhibition. Table 1 summarizes some treatment options based on AhR antagonism and current knowledge of SARS-CoV-2 pathophysiology. 

## 6. Future Research

A number of areas of future research have been highlighted throughout the article. Some of particular importance are listed below. 

AhR activation leads to Synphilin-1 degradation, as shown in neurons [157]. Synphilin-1 regulates AMPK and decreases ACC [78]. ACC leads to the irreversible decarboxylation of acetyl-CoA, suggesting that AhR-induced Synphilin-1 degradation will reduce acetyl-CoA levels. This could indicate that the AhR degradation of Synphilin-1 will modulate the melatonergic pathway, N-ASph, SPMs and acetylated-COX2. This requires clarification in different cell types, especially immune cells, including as to whether AhR degradation of Synphilin-1 is modulated by the acetylation of COX2.

The relevance of racial discrimination stress and associated increase in pro-inflammatory cytokines with suppressed pineal melatonin and increased gut permeability/dysbiosis and kynurenine pathway activity require investigation, given their potential importance in driving the increased levels of SARS-CoV-2 severity/fatality in BAME communities as well as the wider health disparities across race, including the increased severity and poorer outcomes across a wide range of cancers. Wider physiological changes proposed to drive increased BAME susceptibility and severity [158] may interact with the pathways outlined above.

As vitamin D can suppress AhR effects and downstream inductions and decrease gut permeability/dysbiosis [159], the interactions of decreased vitamin D with racial discrimination stress in the regulation of immune responses against COVID-19 and cancers, including via metabolic dysregulation in immune cells, will be important to determine. 

Is there a circadian rhythm to SPM production in immune cells that is mediated by pineal melatonin’s upregulation of acetyl-CoA, therefore allowing OXPHOS and N-ASph to reinvigorating these cells and protecting them from induction into an ‘exhausted’ state? This is supported by data showing an increased cytotoxicity of anti-viral cells in the morning period.

Acute kidney disease is a not uncommon consequence of SARS-CoV-2 infection, with chronic kidney disease a risk factor for SARS-CoV-2-associated fatality [160]. As kidney dysfunction is associated with an increase in indoxyl sulfate (an AhR ligand) [161], it requires investigation as to the role of indoxy sulfate in the suppression of anti-viral responses. The relevance of this in SARS-CoV-2 infection is highlighted by the impact of indoxyl sulfate on the endothelial dysfunction and CVDs associated with fatality in end-stage renal disease, and which is mediated via alterations in T cell function [162], with its detrimental effects, like other AhR agonists, driven by RhoA/ROCK path upregulation in endothelial cells [163]. The role of AhR activation in indoxyl-sulfate-driven increase in platelet pro-coagulative activity [164] and thrombosis [165], including via RhoA/ROCK, will also be important to determine. 

As circulating levels of IL-6 in a sample of older adults (average age 78) correlates with levels of circulating indoxyl sulfate [166], it will be important to determine the role of indoxyl sulfate, via AhR activation, in the pathophysiology of SARS-CoV-2 infection in elderly patients.

The role of increased levels of circulating Aβ42 in the very elderly will be important to investigate in SARS-CoV-2 pathophyisology. As well as activating TLRs, Aβ42 also suppresses the immune-dampening effects of the α7nAChR and SPMs, including via the regulation/suppression of N-ASph and acetylated-COX2. Such investigations are likely to help clarify the role of Aβ42-driven alterations in immune function in the pathophysiology of dementia. 

A number of the diverse catabolites of tryptophan produced in the gut have regulatory effects on the AhR [167]. Processes biasing the gut production of such tryptophan catabolites will be important to determine, including as a consequence of the SARS-CoV-2 virus in the gut [168].

Preclinical data shows that SphK1 can be recruited to mitochondria under stress conditions [169], raising the possibility that acetyl-CoA generated in mitochondria may more readily directly interact with SphK at mitochondria in the regulation of N-ASph formation. This requires investigation in different cell types, given the powerful role for mitochondrial metabolism and acetyl-CoA production in the regulation of N-ASph, SPMs, and acetylated-COX2.

Recent data indicates that air pollutant activation of the AhR can upregulate the ACE2r, which the authors proposed to indicate that the AhR will increase SARS-CoV-2 entry via increased ACE2r availability [170]. As noted, the ACE2r is required to be in lipid rafts and in a dimer, or close association, with other channels/receptors, including B0AT1, suggesting that it is the concurrent upregulation of the ACE2r and its proposed partners by the AhR that is crucial to viral entry in the lung and other organs and tissues. This will be important to experimentally determine.

As SARS-CoV-2 severity is predominately evident in the elderly [171], it is notable that recent data shows the AhR to be increased in the brain with age, being further increased in the brains of Alzheimer’s disease patients, indicating a role for heightened AhR levels in the ageing process [172]. These authors also found the AhR to be increased in the serum of Alzheimer’s disease patients. Many studies have shown the AhR to be relevant to the ageing process, promoting an ageing phenotype across different species, which the authors propose to be mediated via effects on mitochondrial function [173]. The levels of AhR over age in different organs and tissues, and especially in immune cells, will be important to determine, including in correlation with SARS-CoV-2 infection severity. Given the proposed role of the AhR in the regulation of SARS-CoV-2 entry, the ‘cytokine storm’, and anti-viral and anti-cancer defence, factors modulating AhR levels will be important to determine. As indicated above, such factors will include ageing-associated changes in pineal melatonin and gut dysbiosis, as well as stress and dietary and lifestyle factors. 

## 7. Conclusions

The data reviewed above clearly underline the over-looked importance of the AhR in SARS-CoV-2 viral entry and pathophysiology. The three downstream consequences of the ‘cytokine storm’—viz suppressed pineal melatonin, increased gut permeability/dysbiosis and activation of the IDO/kynurenine/AhR pathway—have relevant pathophysiological consequences and are also important to how pre-existent, high-risk medical conditions prime for severity and fatality in SARS-CoV-2 and cancers. The increased SARS-CoV-2 severity/fatality in people from the BAME communities may also be explained by AhR activation arising from racial discrimination stress as well as from economic-status-linked air pollutants. Some key points could be underscored: a) AhR co-ordinates COVID-19 pathophysiology; b) AhR, via mitochondrial metabolism and acetyl-CoA, alters patterned immune responses; c) AhR effects in COVID-19 have overlaps with cancer pathophysiology; d) Racial discrimination stress is proposed to act via the AhR to drive health disparities; e) AhR antagonists may provide prophylaxis and treatment options for COVID-19, cancers, and prevent the biological consequences of racial discrimination stress. The perspective highlighted above has important prophylactic and treatment implications and calls for future research that better integrates physiology with the social nature of the human condition in all its nuanced manifestations.

## Figures and Tables

**Table 1:** Treatment options based on AhR antagonism and current knowledge of SARS-CoV-2 pathophysiology.

Treatment	Mode of Efficacy	References
Vitamin B12/Folate	AhR antagonism; could inhibit entry, cytokine storm and enhance antiviral response	[123]
Vitamin B12	Mpro protease inhibitor; expect to inhibit entry	[124]
	Optimizes NK cell cytotoxicity	[125]
Resveratrol, Curcumin and Rosiglitazone	Inhibitors of the IDO-kynurenine-AhR pathway	[122]
Green Tea’s-Epigallocatechin gallate (EGCG)	AhR antagonist	[127]
- EGCG	MDSC inhibitor; increases CD8+ T cell cytotoxicity	[128]
- EGCG, epicatechingallate and gallocatechin-3-gallate	Mpro antagonist	[133,134]
- EGCG	Inhibits monoamine oxidase; increases 14-3-3 and therefore melatonergic pathway	[135,136]
- EGCG	Induces M2-like macrophage;dampens neutrophils	[140]
- tannic acid	PKM2 inhibitor; enhance NK cell cytotoxicity?	[141]
- caffeine	Adenosine A2A receptor inhibition increases CD8+ T cell and NK cell cytotoxicity	[155]
Melatonin	Inhibits SARS-CoV-2 infection risk	[147,148]
	Increases α7nAChR levels, dampening immune activation	[27]
	Decreases platelet activation	[20]
	Seems to reset immune cell metabolism	[5]
ROCK inhibitors: Y27632	Only Y27632 clinically available but toxicity problematic; efficacy via S1P3r/RhoA/ROCK inhibition	[151]
Curcumin	Acts indirectly to inhibit ROCK pathway	[153]
IDO inhibitors	Potential, but not currently indicated	[154]
Nimesulide	Inhibitor of B0AT1/SLC6A19 and therefore potentially of entry via the ACE2-B0AT1 dimer	[10]

## Abbreviations

α7nAChR	alpha 7 nicotinic acetylcholine receptor
Aβ	amyloid-beta
AANAT	aralkylamine N-acetyltransferase
ACC	acetyl-CoA carboxylase
ACE2r	angiotensin-converting enzyme 2 receptor
acetyl-CoA	acetyl-coenzyme A
AhR	aryl hydrocarbon receptor
BAME	Black Asian and Minority Ethnic
CD8+	cluster of differentiation 8
COVID-19	coronavirus disease-19
COX2	cyclooxygenase 2
CRH	corticotropin-releasing hormone
CVD	cardiovascular disease
CYP	cytochdrome P450
DPP-4	Dipeptidyl peptidase 4
EGCG	epigallocatechin gallate
EP4	prostaglandin E2 receptor 4
HMGB	high-mobility group box
IDO	indoleamine 2,3-dioxygenase
IFN	interferons
IL	interleukin
LPS	lipopolysaccharide
MDSC	myeloid-derived suppressor cells
N-ASph	N-acetylsphingosine
NAS	N-acetylserotonin
NK	natural killer
OXPHOS	oxidative phosphorylation
PDC	pyruvate dyhydroganse complex
PDK	pyruvate dehydrogenase kinase
PGE2	prostaglandin E2
ROCK	RhoA-associated kinase
S1P	sphingosine-1-phosphate
SARS-CoV-2	Severe Acute Respiratory Syndrome associatedcoronavirus
SNP	single nucleotide polymorphism
SOD	superoxide dismutase
SphK	sphingosine kinase
SPM	specialized pro-resolving mediators
TCA	tricarboxylic acid
TCDD	2,3,7,8-tetrachlorodibenzo-p-dioxin
TDO	tryptophan 2,3-dioxygenase
TLR	toll-like receptor
TNF	tumor necrosis factor
